# Observations of the “Egg White Injury” in Ants

**DOI:** 10.1371/journal.pone.0112801

**Published:** 2014-11-13

**Authors:** Laure-Anne Poissonnier, Stephen J. Simpson, Audrey Dussutour

**Affiliations:** 1 Research Center on Animal Cognition, The National Center for Scientific Research and Toulouse University, Toulouse, France; 2 Charles Perkins Centre, The University of Sydney, Sydney, New South of Wales, Australia; University of Sheffield, United Kingdom

## Abstract

A key determinant of the relationship between diet and longevity is the balance of protein to carbohydrate in the diet. Eating excess protein relative to carbohydrate shortens lifespan in solitary and social insects. Here we explored how lifespan and behavior in ants was affected by the quality of protein ingested and the presence of associated antinutrients (i.e. compounds that interfere with the absorption of nutrients). We tested diets prepared with either egg white protein only or a protein mixture. Egg white contains an anti-nutrient called avidin. Avidin binds to the B vitamin biotin, preventing its absorption. First, we demonstrate that an egg-white diet was twice as deleterious as a protein-mixture diet. Second, we show that ingestion of egg-white diet drastically affected social behavior, triggering elevated levels of aggression within the colony. Lastly, we reveal that by adding biotin to the egg white diet we were able to lessen its detrimental effects. This latest result suggests that ants suffered biotin deficiency when fed the egg white diet. In conclusion, anti-nutrients were known to affect health and performance of animals, but this is the first study showing that anti-nutrients also lead to severe changes in behavior.

## Introduction

In insects and mammals it has been shown that a determinant of the relationship between diet and longevity is the balance of protein to carbohydrate [1]. The quantity of dietary protein ingested has been recognized as an important factor for the reduction of lifespan [1], but the nutritional quality of protein is also of critical importance [2]–[5]. Protein quality depends on two main factors: the profile and concentration of amino acids [1], [6]–[7] and the presence of associated compounds that may act as ‘antinutrients’ [8]. Antinutrients have been defined as “substances, which by themselves, or through their metabolic products arising in living systems, interfere with food utilization and affect the health and performance of animals” [9].

In many experiments conducted on omnivorous insect such as ants, eggs white are used as a main source of proteins [10]–[12]. Eggs are considered high quality in their amino acid composition, but the inclusion of large amounts of egg white in special experimental diets causes a definite nutritional disease in animals. This disorder is commonly called egg white injury. Egg whites contain avidin – an antinutrient that reduces longevity of numerous insects [13] mammalian species and fish species [14]. Most insects fed on avidin-containing diets show retarded growth and high mortality rate [13] and cannibalism [15]. Avidin is a glycoprotein, which irreversibly binds biotin and renders it unavailable [16]. Biotin, as a cofactor of major carboxylases, is involved in key process such as gluconeogenesis, lipogenesis, fatty acid and amino acid catabolism, making it essential for insect growth [13].

In this paper, we explored how lifespan and behavior in ants are affected by the quantity of egg white ingested. Ants offer some unique opportunities to tackle this question. First, ants share some fundamental features with other non-social insects. For instance, ants regulate their intake of both protein and carbohydrate when suitable foods are available, but live less long when confined to diets that have an elevated proportion of protein [17]–[21]. Second, ants with a pronounced division of labour between similarly sized sterile worker castes provide a unique opportunity to study the direct effect of egg white ingestion on the evolution of lifespan, independently of reproductive effort [22]. Third, in ants, interactions among individuals coordinate the activities of the entire colony so that it acts as a nutrient acquiring, distributing and digesting ‘superorganism’ [17] and offer the intriguing possibility to consider the relationship between egg white ingestion and social behaviour.

## Methods

### 1- Species studied and rearing conditions

Eight colonies of the black garden ant *Lasius niger*, each comprising ca. 10,000 workers, were collected in Marquefave (South-West France, 43°19′23.7″N 1°15′11.2″E) in 2009 and 2010. No specific permissions were required for these locations/activities. The field studies did not involve endangered or protected species. Experimental colonies of 200 workers without brood were constructed from these mother colonies. In *Lasius niger* nurses are younger than foragers [23]. In order to harmonize the experimental colonies in term of age and task, all workers were collected from the foraging arena rather than within the nest of the mother colonies and were probably all foragers.

Each experimental colony was housed in a plastic box of 100 mm diameter, the bottom of which was covered by a layer of cotton moistened by a cotton plug soaking in a water reservoir underneath. The box was connected to an arena (diameter 150 mm) with walls coated with Fluon to prevent ants from escaping. The nests were regularly moistened and the colonies were kept at room temperature (24–26°C) under a 12∶12 L/D photoperiod. Before starting the experiments, colonies were fed *ad libitum* with honey solution (15%) and prey (mealworms *Tenebrio molitor*) for a week.

### 2- Synthetic foods

In the field, black garden ants scavenge for dead insects, as well as tend and collect honeydew from sap-feeding Homoptera. Accordingly, these ants are confronted with foods varying widely in their ratio of protein to carbohydrate, from near pure sources to mixtures. For the experiment described below, we used synthetic foods varying in the ratio and concentration of protein (P) and digestible carbohydrate (C). The protein content of all the foods consisted of a mixture of casein, whey protein, egg powder, and glucose was used as a digestible carbohydrate source. The quantity of whole egg was kept constant in each food to maintain the same quantity of associated lipid and minerals. Each food contained 0.5% of vitamins (Vanderzant vitamin mixture for insects). The foods were presented to the ants in a 1% agar solution at a 4.5∶1 ratio of agar solution to dry mass of ingredients. Further preparation details are given in [11].

### 3- Experiments

#### Experiment 1: Survival effect of protein type

We investigated if protein source could affect longevity by confining experimental colonies to one of four diets differing in their protein source and in their protein to carbohydrates ratio, with a fixed total P+C of 200 g L^−1^. The two P:C ratios used were 5∶1 and 1∶5. On one hand, we used protein biased diet (5∶1) to amplify any effects of protein quality on variables quantified and on the other hand we used carbohydrate biased diet (1∶5) typically used in most laboratories to rear ants [11]. The diets were either prepared with a mixture of whey protein 18.3%, casein 73.2%, egg yolk 3.1% and egg white 5.4% (1∶5 MIX or 5∶1 MIX) or only with egg white powder 96.9% and egg yolk 3.1% (1∶5 EGG or 5∶1 EGG) (Figure S3 in File S1). The diet 1∶5 EGG was very similar to the most commonly used artificial diet for ant breeding: the chemically undefined Bhatkar and Whitcomb diet [10]. The egg white was pasteurized at a temperature of 56°C for 3–5 min and cooled at 3°C. Since temperatures greater than 70°C are required to denature avidin, a constituent of egg white [24], this was not denatured in our diet. For each synthetic diet, we tested 8 experimental colonies originating from 8 different mother colonies i.e. for each mother colony, one daughter colony was confined to 1∶5 MIX, one to 5∶1 MIX, one to 1∶5 EGG and the last one to 5∶1 EGG. All colonies had ad libitum access to food that was replenished daily. Colonies never collected all the food offered before it was renewed. To assess mortality, the number of dead ants within each colony was counted every day and removed from the colony until all ants had died.

#### Experiment 2: Foraging effects of protein type

We conducted a second experiment to monitor ant foraging behavior. In the first experiment, we showed that an egg-white diet was twice as deleterious as a protein-mixture diet only for the high protein biased diet so we kept only three treatments 1∶5 MIX, 5∶1 MIX and 5∶1 EGG for the following experiment, 1∶5 MIX becoming our “control” for which we expect standard foraging behavior. For each dietary regime, we tested 4 experimental colonies originating from 4 different mother colonies i.e. for each mother colony, one daughter colony was confined to 1∶5 MIX, one to 5∶1 MIX and the last one to 5∶1 EGG. All colonies were starved for 2 days before the beginning of the experiment to encourage food consumption from the first day of the experiment. Afterward, from day 1, all colonies had ad libitum access to food that was replenished daily. The colonies were filmed for 3 hours per day after replenishing the food during 5 days. The film analysis was conducted without knowledge of treatments.

First, we measured mortality rates to ensure that they were comparable to experiment 1. The number of dead ants within each colony was counted every day for 40 days and removed from the colony until all ants died.

Second, we measured colony intake for the first five days of the experiment. The food was placed in the foraging arena in two small containers (diameter, 15 mm; height, 5 mm). The ants had access only to one container; the second was used as a control for measuring and correcting for evaporation. We also provisioned the nest with moistened cotton wool to minimize the water loss. In order to evaluate the colony’s intake, the small containers with the food were weighed within 0.01 mg every day before they were placed in the foraging arena and again after they were removed. We adjusted colony intake to the number of ants in each colony, to take into account differences in mortality between colonies.

Third, we analyzed foraging behavior to quantify how ants responded to each dietary regime. The total number of ants in the foraging arena and the total number of ants feeding were counted every minute for 3 h for five days, providing measures of ‘exploratory behaviour’ and ‘foraging behaviour’ respectively. We also counted the total number of meals eaten over the 3 h recording period for five days and their respective duration. A meal consisted of a contact with the food that lasted more than 10 seconds. Brief contacts with the food (less than 5 seconds) are usually referred as food probing [25].

Lastly, we observed ant inactivity. We extracted from the videos the coordinates of each ant in the foraging arena every second using ImageJ. We assumed that an ant remained inactive when the two following conditions were fulfilled: (1) its displacement between two successive seconds was less than 0.1 mm and (2) the stop duration was at least 10 seconds (corresponding to ten successive observations). The total number of inactive ants in the foraging arena was counted every minute for 3 h for five days. We also recorded the time spent inactive in the foraging arena for each inactive ant.

#### Experiment 3: Behavior effects of protein type

During experiment 1 and 2, we observed that ants were biting each other on the EGG_5∶1 diet in all colonies tested. We conducted a third experiment to specifically monitor ant interactions. We focused our attention on the two protein biased diets (5∶1 MIX and 5∶1 EGG). For each dietary regime we tested 4 experimental colonies originating from 4 different mother colonies i.e. for each mother colony, one daughter colony was confined to 5∶1 MIX and one to 5∶1 EGG. All colonies had ad libitum access to food that was replenished daily. First, the total number of biting events was counted every 3 min for 3 h (after replenishing the food) for five days. Second, at day 6, a pair of ants was placed in a Petri dish (Ø 35 mm, H 10 mm) to observe changes in the behavior of ants with regard to nestmate recognition. We observed the ants for 5 min after their first encounter and classified worker behavior into one of three categories: (i) Indifference: no visible response to one another, (ii) hostility: antagonistic interactions such as mandible gaping, avoidance or intense antennation, (iii) aggression: a physical attack by one or both workers, including lunging, biting, holding or pulling legs or antennae. Each behavior was given an arbitrary score related to increasing aggression: 0, Indifference; 1, hostility and 2, aggression. Overall aggression index for each dyadic encounter was computed as the sum of the aggression scores divided by the total number of scores. To avoid resampling, workers were not returned to their colonies. The ants used represented four treatment combinations, being (i) from the same colony and fed the same diet (N = 12 pairs for both diets 5∶1 MIX and 5∶1 EGG), (ii) from different colonies and fed the same diet (N = 12 pairs for both diets 5∶1 MIX and 5∶1 EGG), (iii) from the same colony and fed different diets (N = 12 pairs for both colonies), or (iv) from different colonies and fed different diets (N = 24 pairs).

#### Experiment 4: Survival and behavior effects of biotin supplementation

Egg white contains avidin – an antinutrient that has been shown to have detrimental effects that are mediated through its biotin-binding activity [13]. Based on the avidin and biotin content of egg white published in [24], [26], we estimated the avidin content of the 5∶1 EGG diet to be 0.4 g.Kg-1 (5.7 µM/L^−1^), the biotin content to be 69 µg.Kg-1 (0.29 µM/L^−1^) and the avidin/biotin ratio on molar basis to be 20∶1. Avidin contains four identical subunits. Each subunit binds one molecule of biotin; thus a total of four biotin molecules can bind a single avidin molecule, i.e the avidin:biotin binding ratio is 1∶4. We investigated if biotin supplementation could affect longevity by confining experimental colonies to the two high protein diets used in the third experiment (5∶1 MIX and 5∶1 EGG) in which we added 0.025 g.Kg-1 of biotin (102.4 µM/L^−1^). As a result, the avidin/biotin ratio on molar basis in both diets became about 1∶20 preventing any biotin deficiency. For each diet we tested eight experimental colonies originating from eight different mother colonies. To assess mortality, the number of dead ants within each colony was counted every day and removed from the colony until all ants died. On day 5, four colonies per treatment were chosen at random and observed for 3 hours and the total number of biting events was counted every 3 min for 3 h (after replenishing the food).

### 4- Statistical Analysis

All statistical tests were conducted with R version 3.1.1. For all experiments, normality was assessed for each variable using a Kolmogorov-Smirnov test. Data were transformed prior to analysis in order to normalize it if needed. For experiment 1, 2 and 4, longevity data across treatment were compared using Cox regression analysis, with protein source, ratio and colony as categorical variables. Protein and ratio were included in the analysis as factors, whereas colony was included as a clustered term (nested factor). For experiment 2, we used linear models with repeated measures to test for the effect of diet and time on intake, proportion of ants in the foraging arena, proportion of foragers feeding, number of meals and proportion of inactive ants. Intakes and proportions were computed using the daily survivors. We used colony of origin as random factor to correct for possible intrinsic behavioral differences among colonies. The time spent feeding (meal duration) and the time spent inactive across treatment were compared using Cox regression analysis, with diet and day as categorical variables. For experiment 3, we conducted a linear model testing how ants’ behavior (aggression index) was affected by the colony of the ant encountered (same or different), the diet of the ant encountered (same or different), and the ant’s own food regime (protein mix or egg protein).

## Results

### Experiment 1: Survival effect of protein type

**Figure 1:**
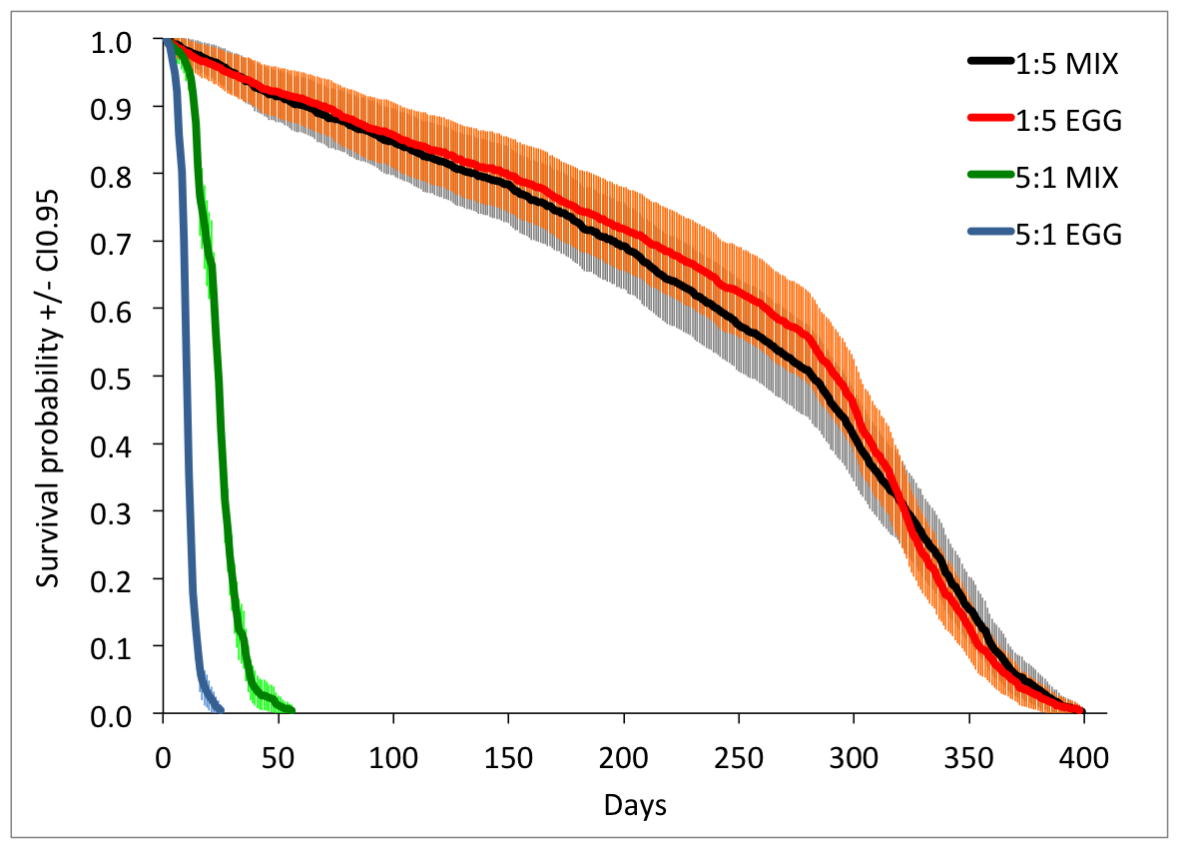
Ant survival according to the protein type and protein to carbohydrate ratio. N = 8 experimental colonies of 200 individuals per treatment. Mortality dynamics were consistent between colonies of the same treatment. The dotted lines represent the confidence intervals.

Ants lived longest on a diet comprising a 1∶5 ratio of protein to carbohydrate and died earlier on a 5∶1 diet (ratio effect Wald = 1701.09, P<0.001). Colonies fed the EGG diet live half as long as colonies fed the MIX diet but only on highly protein biased diets (protein effect Wald = 33.45, P<0.001, protein x ratio effect Wald = 188.58, mean lifespan±CI_0.95_∶243.21±5.28, 248±6.23, 23.34±0.44, 10.06±0.19 days for 1∶5 MIX, 1∶5 EGG, 5∶1 MIX and 5∶1 EGG respectively, Figure 1).

### Experiment 2: Foraging effects of protein type

**Figure 2:**
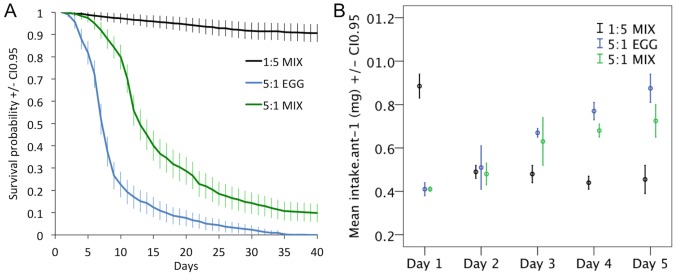
**A.** Ant survival according to protein type and protein to carbohydrate ratio. Mortality dynamics were consistent between colonies of the same treatment. The dotted lines represent the confidence intervals. **B.** Diet intake per ant (colony intake divided by colony size) according to the diet. N = 4 experimental colonies of 200 individuals per treatment. Colony sizes were adjusted to account for ant mortality.

The pattern of longevity observed for experiment 2 was similar to the one observed for experiment 1 (Figure 2A, diet effect Wald = 986.24 P<0.001).

On the high carbohydrate diet (1∶5 MIX) food collection decreased from day 1 to day 2 and remained constant until day 5, whereas on both high protein diets (5∶1 MIX and 5∶1 EGG) food collection increased from day 1 to day 5 (Figure 2B) (diet effect F_2,9_ = 7.08 P = 0.014, day effect F_4,36_ = 16.33 P<0.001, Interaction diet x day F_8,36_ = 39.51 P<0.001). In our previous study conducted in the same exact conditions, we found that the regulation point of protein and carbohydrate intake, known as the intake target, was P:C 1∶4 [18]. Thus, the increase in intake across days observed for both protein diets indicates presumably an effort to reach a certain carbohydrate intake [17]–[18].

**Figure 3:**
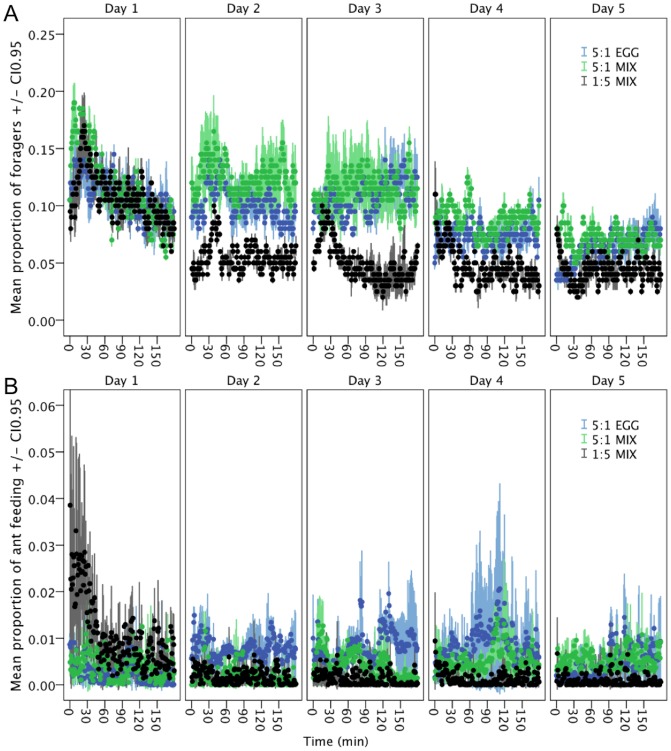
**A** Proportion of ants in the foraging arena according to the diet (number of ants observed in the foraging arena divided by colony size). **B** Proportion of ants in the foraging arena that are feeding according to the diet (number of ants observed feeding divided by colony size). N = 4 experimental colonies of 200 individuals per treatment. Colony sizes were adjusted to account for ant mortality.

The proportion of ants in the foraging arena decreased across days from day 1 for the high carbohydrates diet (1∶5 MIX) and only from day 3 for both high protein diets (5∶1 MIX and 5∶1 EGG) (Table 1, Figure 3A). For the high carbohydrate diet (1∶5 MIX) the proportion of ants in the foraging arena decreased throughout each day while it remained almost constant throughout the day for the high protein diets (5∶1 MIX and 5∶1 EGG).

**Table 1:** Results of a three-way ANOVA to test for the effect of the treatment, the time (1 min to 180 min) and the day (N = 5) at which the measures were done on the proportion of ants in the foraging arena per colony.

Source of variation	Mean squares	df	*F*	*p*
**Between colonies**				
Treatment	0.028	2	16.09	<0.001
**Within colonies**				
Time	0.002	179	7.71	<0.001
Time x Treatment	0.001	358	3.05	<0.001
Day	0.908	4	14.12	<0.001
Day x Treatment	0.146	8	2.28	0.044
Time x Day	0.001	716	7.03	<0.001
Time x Day x Treatment	0.001	1432	3.50	<0.001

The proportion of ants feeding differed according to the diet received (Table 2, Figure 3B). For the high carbohydrate diet (1∶5 MIX), the proportion of ants feeding decreased strongly from day 1 to day 2 and then remained constant. When the high carbohydrate diet (1∶5 MIX) was introduced to the colony on day 1, the number of ants feeding increased exponentially over the first hour, indicating a strong recruitment process due to the food deprivation experienced before, and then decreased. This pattern was not observed during the following days because the colonies were fed ad libitum from day 1. By contrast, the proportion of ants feeding remained constant throughout each day for both high protein diets (5∶1 MIX and 5∶1 EGG) but increased from day to day, presumably in an effort to maintain a constant carbohydrate intake.

**Table 2:** Results of a three-way ANOVA to test for the effect of the treatment, the time (1 min to 180 min) and the day (N = 5) at which the measures were done on the proportion of ants feeding per colony.

Source of variation	Mean squares	df	*F*	*p*
**Between colonies**				
Treatment	0.008	2	9.43	<0.001
**Within colonies**				
Time	0.0005	179	2.78	<0.001
Time x Treatment	0.0006	358	3.63	<0.001
Day	0.002	4	1.39	0.257
Day x Treatment	0.009	8	6.33	<0.001
Time x Day	0.0004	716	4.34	<0.001
Time x Day x Treatment	0.0002	1432	2.87	<0.001

**Figure 4:**
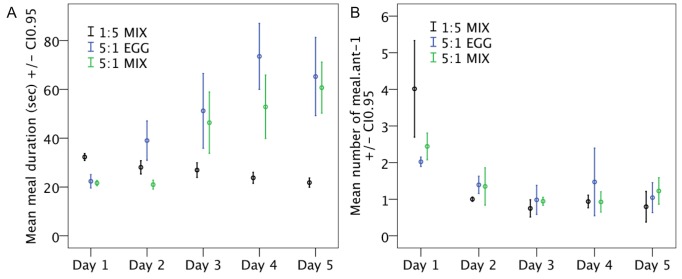
**A.** Meal duration (in seconds) according to the diet. **B.** Relative number of meals per ant (total number of meals divided by colony size). N = 4 experimental colonies of 200 individuals per treatment. Colony sizes were adjusted to account for ant mortality.

For both high protein diets, the difference observed in proportion of ant feeding was due to a difference in meal duration (Figure 4A) and not to a difference in the absolute number of meals (Figure 4B), while it was the opposite for the high carbohydrate diet. The relative number of meals was higher on the first day especially on the high carbohydrate diet (1∶5 MIX) reflecting food deprivation experienced the day before (Figure 4B, diet effect F_2,9_ = 0.09 P = 0.919, day effect F_4,36_ = 94.67 P<0.001, Interaction diet x day F_8,36_ = 13.63 P<0.001). Meal duration increased across days only for the high protein diets (5∶1 MIX and 5∶1 EGG, diet effect Wald = 157.73 P<0.001, day effect Wald = 52.35 P<0.001, Interaction diet x day Wald = 332.46.14 P<0.001, Figure 4A, Figure S1 in File S1), by contrast it remained constant for the high carbohydrate diet (1∶5 MIX).

**Figure 5:**
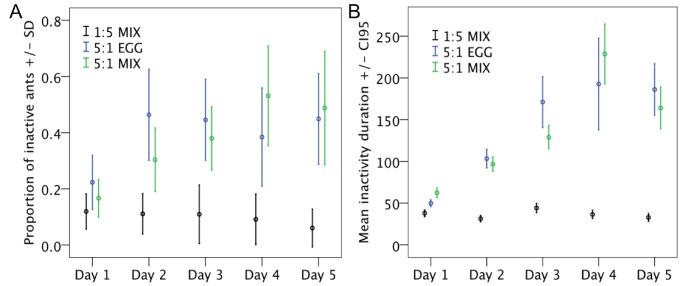
**A.** Proportion of inactive ants in the foraging arena according to the diet (total number of inactive ants observed divided by the number of ants observed in the foraging arena). **B.** Inactivity duration (in seconds) according to the diet. N = 4 experimental colonies of 200 individuals per treatment. Colony sizes were adjusted to account for ant mortality.

From day 2, the proportion of inactive ants in the foraging arena was significantly higher for both high protein diets (5∶1 MIX and 5∶1 EGG) than for the high carbohydrate diet (1∶5 MIX) (Figure 5A, Table 3). The time spent inactive increased for both high protein diets (5∶1 MIX and 5∶1 EGG) while it remained constant for the high carbohydrate diet (diet effect Wald = 70.51 P<0.001, day effect Wald = 23.95 P<0.001, Interaction diet x day Wald = 82.32 P<0.001, Figure 5B, Figure S2 in File S1). On both high protein diets we observed about ten percent of ants that were inactive for long periods of time, from 10 min to 3 hours (Figure S2 in File S1). In contrast, on high carbohydrate diet no ants were observed inactive for more than 10 min (Figure S2 in File S1). Ants observed motionless for long periods of time looked moribund (slight curling of the antennae and legs).

**Table 3:** Results of a three-way ANOVA to test for the effect of the treatment, the time (1 min to 180 min) and the day (N = 5) at which the measures were done on the proportion of inactive ants.

Source of variation	Mean squares	df	*F*	*p*
**Between colonies**				
Treatment	898.51	2	42.64	<0.001
**Within colonies**				
Time	0.070	179	10.95	<0.001
Time x Treatment	0.029	358	4.58	<0.001
Day	10.16	4	10.74	<0.001
Day x Treatment	6.42	8	6.79	<0.001
Time x Day	0.022	716	3.26	<0.001
Time x Day x Treatment	0.022	1432	3.36	<0.001

### Experiment 3: Behavior effects of protein type

Ants were observed fighting almost only on the 5∶1 EGG diet and the occurrence of this aggressive behavior increased across subsequent days (Diet effect F_1,6_ = 78.72, P<0.001, Day effect F_4,24_ = 20.96 P<0.001, interaction diet x day F_4,24_ = 19.09 P<0.001).

**Figure 6:**
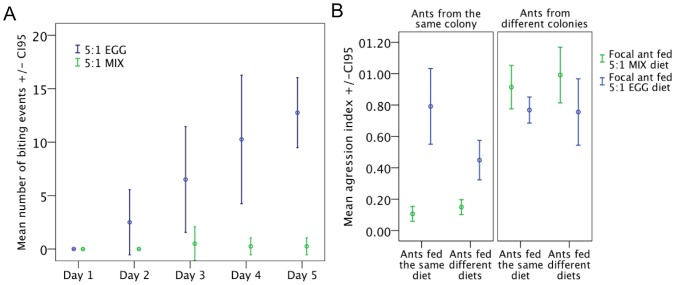
**A** Mean number of biting events observed. Nests were scanned 60 times (every 3 min for 3 hours) for 5 days. **B** Mean aggression index (see text for definition) of workers fed with a mix of protein (5∶1 MIX, green error bars) or egg protein (5∶1 EGG, Blue error bars) encountering workers coming from the same or a different colony fed the same or a different diet. N = 4 experimental colonies of 200 individuals per treatment.

Ants fed the 5∶1 EGG diet were much more likely to fight with nestmates than ants fed the 5∶1 MIX diet, which were never observed to be aggressive towards a nestmate (Table 4, Figure 6B). This effect was slightly more pronounced if the nestmate was also fed the 5∶1 EGG diet (Table 4, Figure 6B). As expected in *Lasius niger* [27], all ants responded aggressively towards non-nestmates, no matter the diet they were fed (Table 4, Figure 6B).

**Table 4:** Linear model testing how an ant’s behaviors during an encounter with another ant (aggression index) was affected by the colony of the ant encountered (same or different), the diet of the ant encountered (same or different) and the ant’s own food regime (treatment: protein mix or egg protein).

Source of variation	Mean squares	df	*F*	*p*
**Colony (same or different)**	11.21	1, 184	88.42	**<0.001**
**Diet (same or different)**	0.16	1, 184	1.30	0.255
**Treatment (MIX or EGG)**	1.09	1, 184	8.60	**0.004**
Colony * Diet	0.40	1, 184	3.13	0.079
Colony * Treatment	5.60	1, 184	44.17	**<0.001**
Diet * Treatment	0.68	1, 184	5.33	**0.022**
Colony * Diet * Treatment	0.26	1, 184	2.08	0.150

### Experiment 4: Survival and behavior effects of biotin supplementation

**Figure 7:**
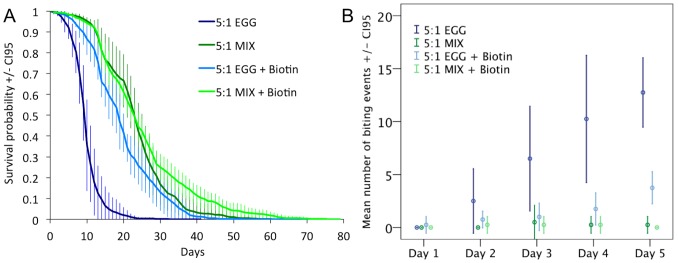
**A** Ant survival according to the protein type used to prepare the high-protein diet and the supplementation in biotin. N = 8 experimental colonies of 200 individuals per treatment. Mortality dynamics were consistent between colonies of the same treatment. **B** Mean number of biting events observed. Nests were scanned 60 times (every 3 min for 3 hours) for 5 days. N = 4 experimental colonies of 200 individuals per treatment.

Life expectancy was increased when we added biotin to the 5∶1 EGG diet (Biotin effect, Wald = 133.82, P<0.001, mean lifespan±CI_0.95_∶19.78±0.19 and 9.53±0.19 for 5∶1 EGG+Biotin and 5∶1 EGG, Figure 7A) but not when we added biotin to the 5∶1 MIX diet (interaction Treatment x Biotin, Wald = 94.21, P<0.001, mean lifespan±CI_0.95_∶25.29±0.70 and 23.34±0.44 for MIX+biotin and MIX, Figure 7A). Nevertheless, ants lived longer on the 5∶1 MIX diet supplemented with biotin than on the 5∶1 EGG diet supplemented with biotin (Wald = 36.19, P<0.001, Figure 7A). Ants fed the 5∶1 EGG+Biotin or the 5∶1 MIX+Biotin diet were rarely observed to be aggressive towards a nestmate (Biotin effect F_1,12_ = 39.15, P<0.001, Interaction Biotin x diet F_1,12_ = 37.58 P<0.001, Figure 7B).

## Discussion

The main aim of this study was to investigate how protein quality and quantity affects longevity and behavior in ants. First we showed that lifespan was reduced when ants were fed with high-protein, low- carbohydrate diets. Second, we found that when we modified the protein source of our diets, egg white protein was more deleterious than a mix of various proteins. Third, we showed that being fed egg white protein in large quantity profoundly affects behavior, triggering intra nest aggression. Lastly, we revealed that being fed biotin in addition to egg white protein nearly doubled longevity and decreased intra-nest aggression. This latest results suggests that avidin an anti-nutrient present in large quantity in egg white, binds biotin and as a consequence ants suffers from biotin deficiency.

Five hypotheses could be proposed to explain the reduction in survival observed on the 5∶1 EGG diet when compared to the 5∶1 MIX diet.

First, such a longevity decrease could result from a deficiency or imbalance of amino acids in the food. However a mixture of egg white powder and yolk contain all the essential and non-essential amino acids in ratios and concentrations that make this unlikely as an explanation (Figure S3 in File S1).

Second, were there an increase in amount of food ingested on 5∶1 EGG diet, this might explain the decrease in survival probability [18]. The key determinant of the relationship between diet and longevity in ants and other insects is the balance of protein and non-protein energy ingested [1], [17]–[21]. Lifespan is reduced when ants ingest high quantity of protein. However we did not observe any difference between 5∶1 EGG and 5∶1 MIX in the colony intake. The only differences were between the high carbohydrate diet (1∶5 MIX) and the high protein diets (5∶1 MIX and 5∶1 EGG). Colonies fed with the high-protein diets collected substantial excesses of food relative to the amount collected on the high carbohydrate diet. The only exception was the very high intake on the high carbohydrate diet seen on day 1. It reflected a combination of food deprivation experienced the day before [17]–[18], [28] and the high phagostimulant efficiency of the high carbohydrate diet in comparison to the high protein diets [29]. After day 1, the intake on the high carbohydrate intake remained constant while the intake on high protein diets increased. In previous studies, we have established that colonies are able to switch between two nutritionally imbalanced but complementary foods to maintain the supply of protein and carbohydrate [17]–[18]. In this study, we explored the responses of colonies when confined to a single diet containing an excess of nutrient relative to the other. Therefore, the ants were forced to ingest foods that were imbalanced and confronted the situation wherein there is conflict between meeting their requirements for protein and carbohydrates. In all studies published so far in ants, it was shown that colonies without larvae collect substantial excesses of protein when fed with high protein diet in an effort to maintain a constant carbohydrate intake [17]–[21].

Third, were there an increase in effort spent in foraging activities on 5∶1 EGG diet, this might explain the decrease in survival probability [30]. However we did not observe any differences between 5∶1 EGG and 5∶1 MIX in the number of ants exploring or feeding. The only differences were again between the high carbohydrate diet (1∶5 MIX) and the high protein diets (5∶1 MIX and 5∶1 EGG). On the first day of the experiment, after two days without food, the number of ants exploring and feeding at the high carbohydrate diet increased exponentially during the first hour due to food deprivation experience beforehand, but decreased thereafter, indicating that the colony reached satiety [31]. This pattern was not observed the following days as food was renewed every day. Consequently the foraging activity on the high carbohydrate diet was relatively high on day 1, decreased on day 2 and remained stable. In contrast the number of ants in the foraging arena remained relatively high throughout the days on both high protein diets. Interestingly, these ants were not observed feeding on day 1, but instead appeared to be engaged in ‘exploratory’ behavior. The number of ant feeding increased from day 2, reflecting again an attempt for compensating the lack of carbohydrate in the high protein diet [17], [18].

Fourth, a difference in protein toxicity may explain the reduction in survival observed on the 5∶1 EGG diet when compared to the 5∶1 MIX. The level of toxicity can be expressed in term of level of sickness. It has been shown that moribund or sick ants [32], [33] and honeybees [34] leave their nest and die in isolation. In our experiment, on both high protein diets, numerous ants were observed motionless and moribund close to the edge of the foraging arena, from day 2 for the 5∶1 EGG diet and from day 3 for the 5∶1 MIX diet. In contrast we did not observe any moribund ant on the high carbohydrate diet (1∶5 MIX). It has been shown before that high-protein diets increase mortality rates in ants and other insect [1], [17]–[21], but it remains unclear how. Several explanations have been posed to explain protein toxicity. First, elevated levels of proteins increase the target of rapamycin (TOR) pathway signaling which in turn activate aging processes in mice [35], but it is not yet proven in insect. Second, protein toxicity could result from the limited ability to digest proteinaceous foods in ants [36]–[39]. Lastly, life-shortening effects of protein could be linked to the elimination of nitrogenous waste. Nevertheless, this does not explain why the 5∶1 EGG diet affected the ant earlier than the 5∶1 MIX diet. The toxicity of the EGG diet might have been increased by the presence of avidin – an antinutrient in egg, which irreversibly binds biotin and makes it unavailable. Following this hypothesis, ants were dying earlier on an EGG diet in part as result of biotin deficiency or its consequences. The last experiment where ants were fed with EGG diet supplemented with biotin supports this hypothesis. Dietary studies that have added extra biotin back to the diet have allowed insects to overcome the presence of avidin [40]. Interestingly, in honeybees, another social hymenoptera, avidin ingestion had no significant impacts on longevity [41], [42]. However the concentration in avidin used in [41], [42] was 5 times lower than the concentration in our EGG diet.

Lastly, decreased longevity might have resulted from behavioral modification during interactions. It has been shown that sick ants increase their level of aggression [33]. Following this hypothesis, ants were dying in part as result of elevated level of aggression or its consequences. The third experiment where ants were observed biting each other only on the 5∶1 EGG diet supports this hypothesis. As mentioned in Bos et al [33] “self- removal and increased aggression are reminiscent of what is also found in humans, where sick individuals become reclusive and irritable, isolating themselves from other individuals”. Interestingly, biotin supplementation limited ants aggression. Two hypotheses could be advanced to explain the link between biotin deficiency and aggression level. First, in insects, certain cuticular hydrocarbons are synthesized from fatty acid via the elongation-decarboxylation pathway [review in 43]. Because biosynthesis of fatty acid depends in part on biotin [44], the hydrocarbon profiles of ants fed on an egg white diet might have been altered, compromising ant recognition and explaining the aggressive behaviour observed between congeners. However we did not find any evidence for a link between biotin and hydrocarbons synthesis in the literature. Analysis of hydrocarbons profile will be needed to corroborate this hypothesis. Since the brain is quite vulnerable to biotin deficiency [45], the second hypothesis is that biotin deficiency might have led to cognitive impairment and limited ant recognition performance. In rats for example, biotin deficiency produces neurological symptoms that range from ataxia to sensory loss [46].

The question we might ask is why ants ate such large quantities of toxic high protein diet. To this point of the paper we have focused our interpretations on the macronutrients – protein and carbohydrates and stated that ants collected excesses of protein in an effort to acquire a certain carbohydrate intake. As the literature shows [1], macronutrients can explain a good deal of the variation in the behavioral, physiological and performance responses of animals. Macronutrients are, however, clearly not the only functionally important nutritional components of foods: vitamins and minerals are essential to health and also play a critical role in an animal’s nutritional strategies. Being able to determine the presence and concentrations of nutrients in foods by taste is clearly advantageous, but not all nutrients in food are detected by specialized taste receptors [1]. This is particularly the case for micronutrients such as vitamins, which are essential to health but required in very small amounts relative to the macronutrients protein and carbohydrate [1]. If the nutritional consequences of eating a food are not apparent until after a meal is processed and absorbed. The animal has to learn from the experience of having eaten a food by associating post-ingestive nutritional consequences (biotin deficiency) with properties of the food, however this take some times as biotin deficiency is not instantaneous but is steadily rising from day to day [13]. In our study, ants confined to an egg white diet, might increase their intake from day to day to also provide limiting biotin, constraining them as a result to ingest more avidin and enter a deadly loop. Here, we have shown that in ants, the effects of biotin deficiency are not limited to health and performance, but extend to behavior modification and by extension to social organization. Knowing that egg white protein is a common component of ant diets in many laboratories, it might be important to add biotin to synthetic diets to improve ant husbandry.

Biotin is a coenzyme required for all forms of life, feeding avidin or streptavidin causes a biotin deficiency that leads to stunted growth and mortality in numerous insect [16], [47] to which we can now add ants. Proteins that bind to vitamins, such as biotin, had been shown to represent potential pest-resistance transgene products [40]. The avidin gene has recently been incorporated into genetically modified crop plants, which are then insecticidal to a variety of insects [13], [16] and aphids [40]. However, many aphid pests of major plant crops are attended or attacked by ants. In light of our results, it might be important to look at the ecological significance of aphids as carriers of avidin from transgenic plants to ant colonies.

## Supporting Information

File S1 Includes Figures S1–S3. **Figure S1:** Natural logarithm of the fraction of ants that are still feeding as a function of time from day 1 to day 5. Note that each plot corresponds to the survival curve of more than 600 meal durations. If the probability for an ant to stop feeding was constant over time then the log-survival curve of the number of ants still feeding should fit a straight line (Haccou and Meelis, 1992). However, from day 2 to day 5, for the high protein diets (5∶1 MIX and 5∶1 EGG) the curves suggest that the duration of a meal was either short or long. **Figure S2:** Natural logarithm of the fraction of ants that are still inactive as a function of time from day 1 to day 5. Note that each plot corresponds to the survival curve of more than 800 stop durations. If the probability for an ant to initiate a new displacement was constant over time then the log-survival curve of the number of ants still inactive should fit a straight line (Haccou and Meelis, 1992). However, from day 2 to day 5, for the high protein diets (5∶1 MIX and 5∶1 EGG) the curves suggest that the duration of a stop was either short or long. **Figure S3**: Amino acid profile of both the MIX diet and the EGG diet. The MIX diet and the EGG diet were prepared with the following protein sources: whey protein 18.3%, casein 73.2%, egg yolk 3.1% and egg white 5.4% (MIX); egg yolk 3.1% and egg white 96.9% (EGG).
(DOCX)
