# Supplementary material for: Observations of the “Egg White Injury” in Ants
Source: PLoS One. 2014 Nov 13;9(11):e112801. doi: 10.1371/journal.pone.0112801 (PMC4231089; doi:10.1371/journal.pone.0112801)
Supplement: File S1 — Includes Figures S1–S3. Figure S1: Natural logarithm of the fraction of ants that are still feeding as a function of time from day 1 to day 5. Note that each plot corresponds to the survival curve of more than 600 meal durations. If the probability for an ant to stop feeding was constant over time then the log-survival curve of the number of ants still feeding should fit a straight line (Haccou and Meelis, 1992). However, from day 2 to day 5, for the high protein diets (5∶1 MIX and 5∶1 EGG) the curves suggest that the duration of a meal was either short or long. Figure S2: Natural logarithm of the fraction of ants that are still inactive as a function of time from day 1 to day 5. Note that each plot corresponds to the survival curve of more than 800 stop durations. If the probability for an ant to initiate a new displacement was constant over time then the log-survival curve of the number of ants still inactive should fit a straight line (Haccou and Meelis, 1992). However, from day 2 to day 5, for the high protein diets (5∶1 MIX and 5∶1 EGG) the curves suggest that the duration of a stop was either short or long. Figure S3: Amino acid profile of both the MIX diet and the EGG diet. The MIX diet and the EGG diet were prepared with the following protein sources: whey protein 18.3%, casein 73.2%, egg yolk 3.1% and egg white 5.4% (MIX); egg yolk 3.1% and egg white 96.9% (EGG). (DOCX) [file pone.0112801.s001.docx]

Ffi


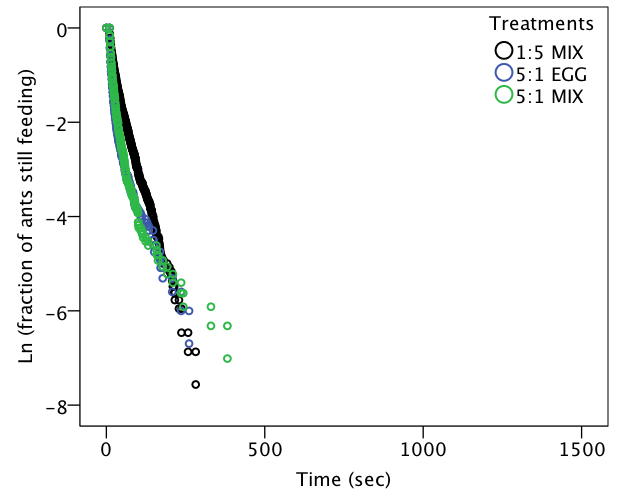

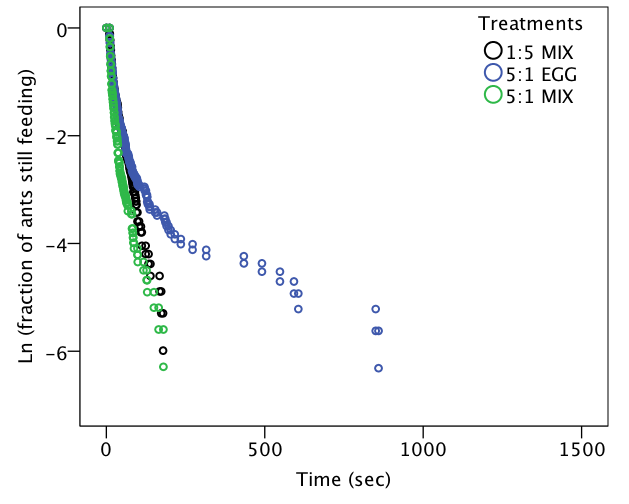

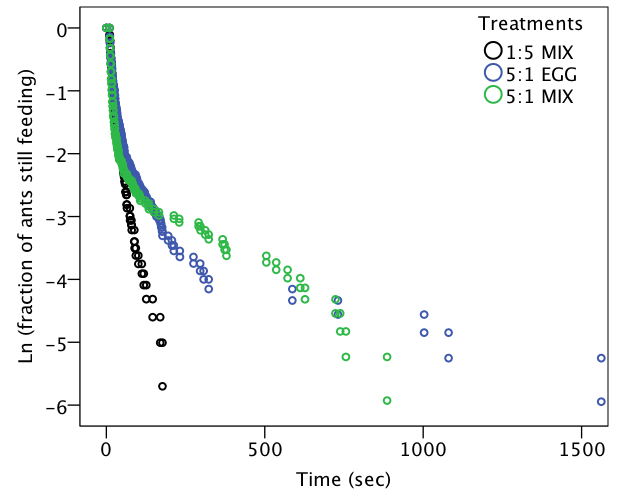

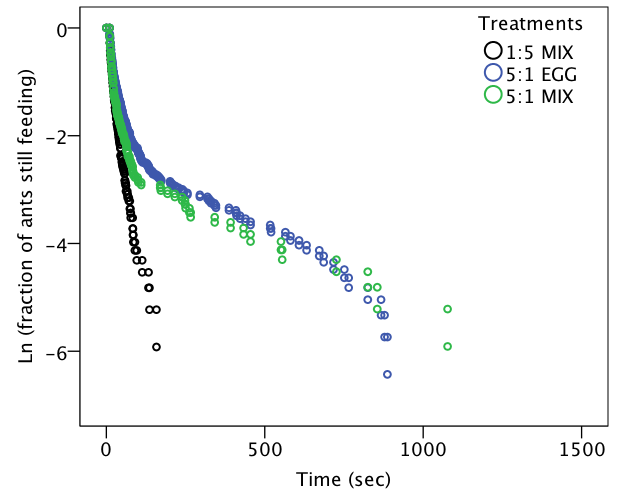

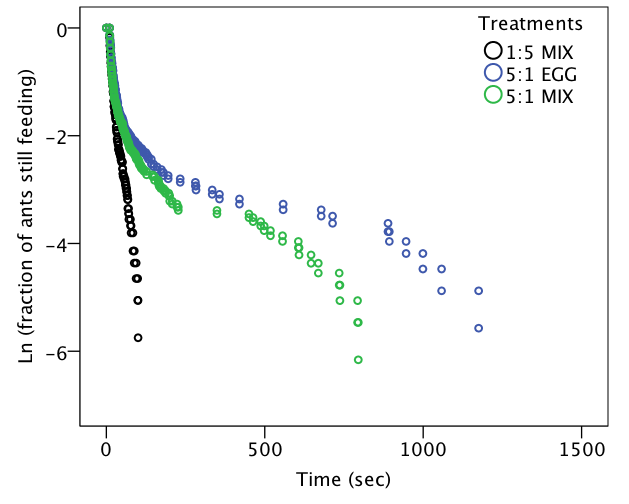


**Figure S1**: Natural logarithm of the fraction of ants that are still feeding as a function of time from day 1 to day 5. Note that each plot corresponds to the survival curve of more than 600 meal durations. If the probability for an ant to stop feeding was constant over time then the log-survival curve of the number of ants still feeding should fit a straight line (Haccou and Meelis, 1992). However, from day 2 to day 5, for the high protein diets (5:1 MIX and 5:1 EGG) the curves suggest that the duration of the meals was either short or long.


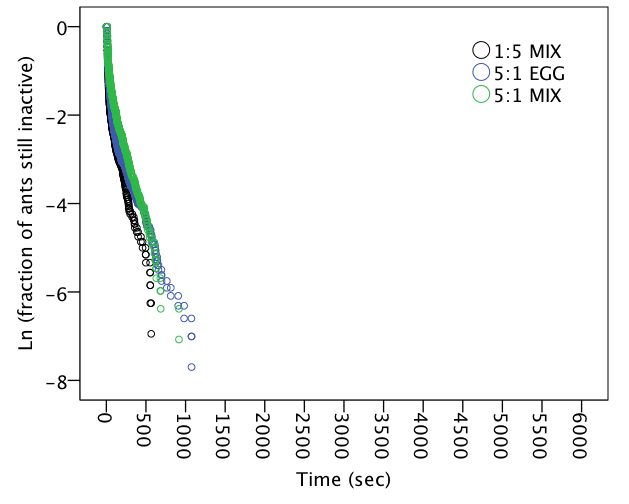

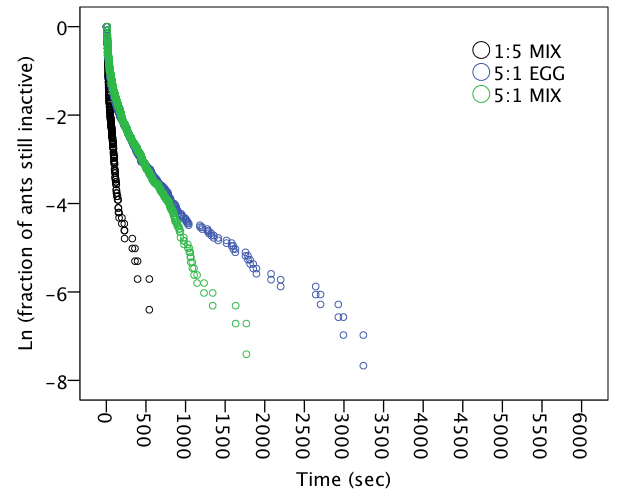

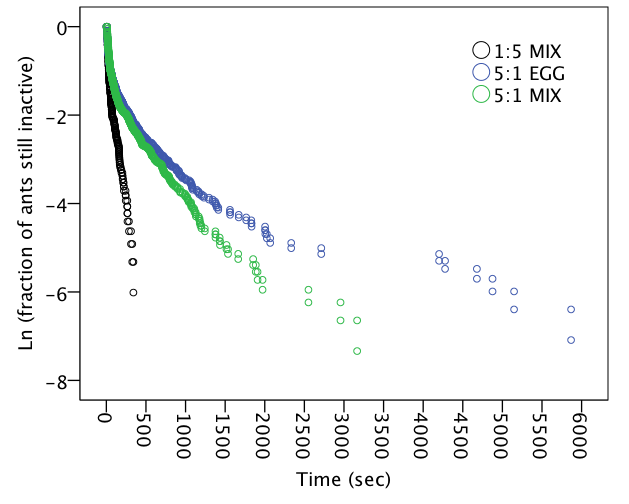

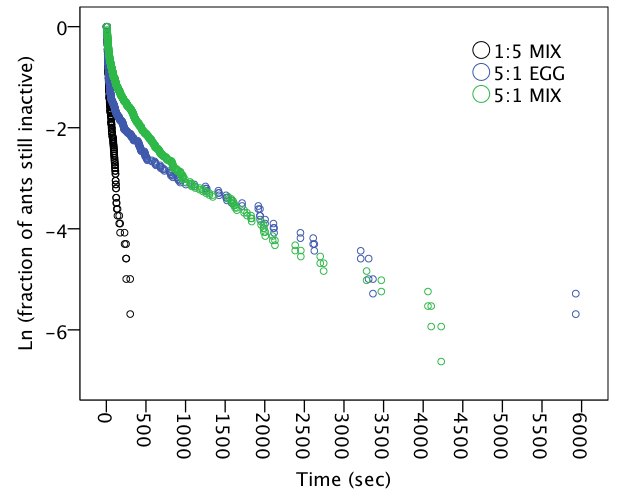

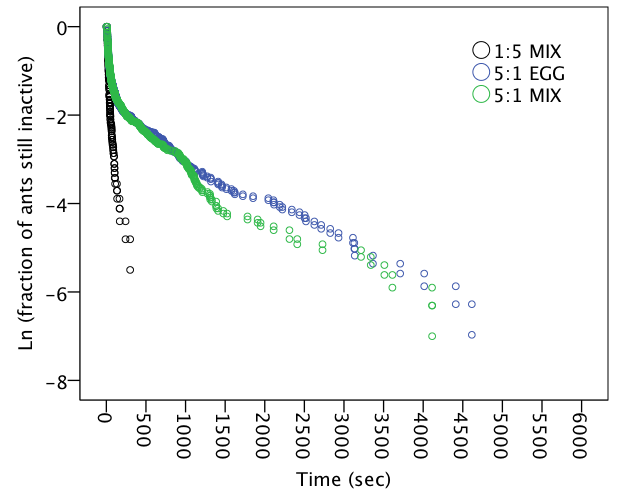


**Figure S2:** Natural logarithm of the fraction of ants that are still inactive as a function of time from day 1 to day 5. Note that each plot corresponds to the survival curve of more than 800 stop durations. If the probability for an ant to initiate a new displacement was constant over time then the log-survival curve of the number of ants still inactive should fit a straight line (Haccou and Meelis, 1992). However, from day 2 to day 5, for the high protein diets (5:1 MIX and 5:1 EGG) the curves suggest that the duration of the stops was either short or long.


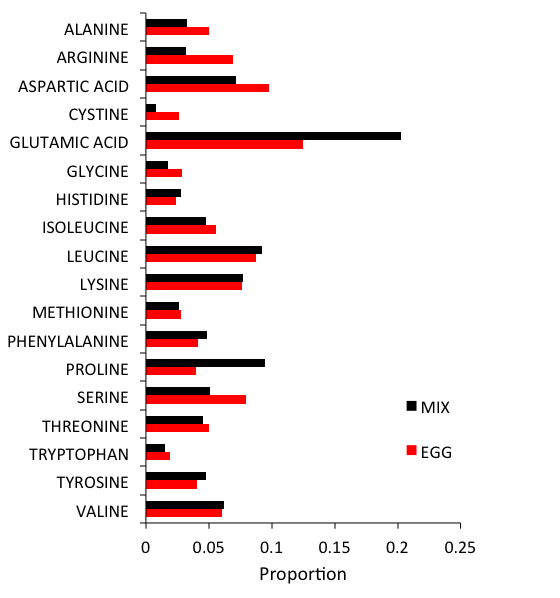


**Figure S3**: Amino acid profile of both the MIX diet and the EGG diet. The MIX diet and the EGG diet were prepared with the following protein sources: whey protein 18.3%, casein 73.2%, egg yolk 3.1% and egg white 5.4% (MIX); egg yolk 3.1% and egg white 96.9% (EGG).
